# Ketosis and bipolar disorder: controlled analytic study of online reports

**DOI:** 10.1192/bjo.2019.49

**Published:** 2019-07-04

**Authors:** Iain H. Campbell, Harry Campbell

**Affiliations:** PhD student, Usher Institute of Population Health Sciences and Informatics, University of Edinburgh, UK; Professor of Genetic Epidemiology and Public Health, Usher Institute of Population Health Sciences and Informatics, University of Edinburgh, UK

**Keywords:** Bipolar disorder, ketogenic diet, ketosis

## Abstract

**Background:**

Members of online bipolar disorder forums often report experiences of mood-stabilisation on the ketogenic diet, which has traditionally been used in the treatment of epilepsy. We examined the nature and extent of such reports.

**Aims:**

To investigate associations between a ketogenic diet and mood stabilisation among individuals with bipolar disorder.

**Method:**

We undertook an observational analytic study of free-text comments in online forums about mood effects of dietary interventions (ketogenic, omega-3 enriched or vegetarian) classified by *a priori* categories of change in mood stabilisation in 274 people with bipolar disorder.

**Results:**

There were 141 (85.5%) free-text comments on ketogenic diets that reported a positive impact on mood stabilisation. Reports of significant mood stabilisation or remission of symptoms over a period were substantially higher for a ketogenic diet than for other diets (93/165, 56.4%, 95% CI 48.4–64.1) *v.* 14/94, 14.9%, 95% CI 8.4–23.7), odds ratio 7.4, 95% CI 3.8–14.1, *P* < 0.0001), many with detailed reports of the improvements experienced and several lasting for extended periods (months to years). Other reported associations included fewer episodes of depression (in 41.2%, 95% CI 30.6–52.4 of individuals); improved clarity of thought and speech (28.2%, 95% CI 19.0–39.0); increased energy (25.9, 95% CI 17.0–36.5); and weight loss (25.9%, 95% CI 17.0–36.5).

**Conclusions:**

Despite the inherent limitations of the observational data based on self-reports posted online, the association strength and reports of sustained benefit support a hypothesis of a ketogenic diet being associated with beneficial effects on mood stabilisation. Caution should be exercised in interpreting this data until a controlled trial can be carried out to examine this hypothesis. These preliminary observations are generally consistent with a mitochondrial dysfunction component to bipolar disorder aetiology with ketones bypassing a block between glycolysis and the tricarboxylic acid cycle.

**Declaration of interest:**

None.

A ketogenic diet is currently indicated as an effective, non-pharmacological treatment for medically resistant epilepsy and can result in long-term seizure reduction.^1,2^ Ketosis is a natural metabolic state experienced in the fasted condition in which the primary source of metabolic fuel for mitochondrial adenosine triphosphate (ATP) production switches from glucose to ketone bodies derived from breakdown of fatty acids. Ketosis induced by a ketogenic diet results in alteration of neurotransmitter levels, hormones and peptides^3,4^ and has mitochondrial effects including increased oxidative phosphorylation,^5^ increased ATP production,^5^ increased glutathione levels,^6^ reduced reactive oxygen species production^7^ and increased mitochondrial biogenesis.^8^ The ketotic state is also neuroprotective under conditions of oxidative stress.^9^

Recent research data have resulted in increasing attention being given to the hypothesis of mitochondrial dysfunction having a causal role in bipolar disorder and this has recently been elaborated in detail.^10^ Considering the profound mitochondrial effects of the state of ketosis we conducted an initial literature review of bibliographical databases to identify clinical case reports or trials/quasi-experimental studies on the impact of a ketogenic diet on mood stabilisation in bipolar disorder. This yielded only two small reports.^11^ In the first study ketone bodies were not detected in the urine of the participant, indicating a failure to achieve a state of ketosis.^12^ This is likely because of the restrictive nature of the diet. The second study comprised case reports of two women who did achieve ketosis for 2–3 years with the participants experiencing significant mood stabilisation, which they reported as exceeding that achieved with medication. One participant reported that ‘being in ketosis has been life changing for me’.^11^ Given the demonstrated effectiveness of a ketogenic diet in refractory epilepsy and the parallels between many aspects of bipolar disorder and epilepsy, we hypothesised that a ketogenic diet might have a role in mood stabilisation in bipolar disorder. We then conducted an observational analytic study of free-text comments in online forums about the mood effects of dietary interventions from individuals with bipolar disorder (self-reported) to conduct a preliminary investigation of reported associations between a ketogenic diet and mood stabilisation to assess whether there was any support for this hypothesis that may be worth investigating further.

## Method

### General approach

Text-mining techniques have become an accepted method for the evaluation of public experience in online forums^13,14^ and as a useful tool for hypothesis generation. Since there are several websites with large followings that are dedicated to bipolar disorder we decided to employ text mining to investigate reports of mood stabilisation associated with adoption of a ketogenic diet. To account for reporting biases in online forums we adopted an observational analytic study design with a ‘control’ group by comparing reports of ketogenic diet with similar reports for other ‘control’ dietary interventions (omega-3 enriched or vegetarian) that have been proposed and reported on in the same online forums. We classified entries according to predefined response categories ranging from ‘remission of symptoms’ to ‘significant deterioration in symptoms’ (see below). In as far as this was possible, we checked for duplicate reports and excluded these so that all reports are from separate people. To minimise observation bias we scored online entries using response category definitions that we defined *a priori* (see supplementary File 1 for *a priori* criteria/definitions adopted for classifying online entries; available at https://doi.org/10.1192/bjo.2019.49) and we conducted parallel independent scoring that was partially masked to diet category (the first reviewer prepared edited versions of online posts for the second reviewer with dietary details redacted to the extent that this was possible). Finally, to control for ‘herd’ effects in specific online forums we sought independent replication of findings in different online resources: in a large bipolar disorder online forum (study 1) and by data mining other online bipolar disorder forums (study 2).

### Online bipolar disorder forums

We performed an online search using Google.com to find forums on bipolar disorder with large memberships. We identified the ten forums with the largest memberships.

### Search strategy

We developed a Python text-mining script to trawl online forums for bipolar disorder. The script retrieved posts across all forums that mentioned ‘bipolar disorder’ or ‘manic depression’ in co-occurrence with ‘ketogenic diet’ or synonyms ‘Keto’ and ‘LCHF’ (Low Carb High Fat). We then used the text-mining script to repeat the exercise for two control key phrases. The first ‘omega 3’ is a researched and widely recommended dietary supplement for bipolar disorder. The second ‘vegetarian’, which is a diet with no formal research in relation to effects on bipolar disorder.

### Classification of entries describing effects on mood

We filtered all posts to identify those that mentioned a direct experience of an individual reported to have bipolar disorder when following a ketogenic diet. These posts were identified through occurrence of first-person pronouns. Six ‘response categories’ covering the range of possible outcomes from remission of symptoms to significant deterioration in symptoms/mood were defined *a priori* to guide classification. The posts were then rated by two independent reviewers (I.C. and H.C.) on a categorical scale (see below). The second reviewer was masked to diet group status by redaction of text identifying diet group, where this was possible. Any differences in classification were resolved through detailed discussion of selected online posts. We excluded multiple comments from the same individual.

- Remission of symptoms and/or discontinuation of bipolar disorder medications with stable mood. Criteria: post reports complete mood stabilisation with no depressive/manic episodes while on diet and/or discontinuing bipolar disorder medication.
- Significant improvement in mood stabilisation. Criteria: post reports a much higher level of mood stabilisation to that experienced prior to adopting the diet.
- Some improvement in mood stabilisation. Criteria: some mood stabilisation reported.
- No difference in mood stabilisation noted. Criteria: no difference in mood reported.
- Some deterioration in mood destabilisation. Criteria: some mood destabilisation reported.
- Significant deterioration in mood destabilisation. Criteria: post reports a far higher level of mood destabilisation to what they have previously experienced before adopting the diet.
- Admission to hospital or care-seeking for deterioration of bipolar disorder and/or quality of life. Criteria: post reports admission to hospital or care-seeking as a direct result of adopting the diet.

### Plausibility of bipolar disorder diagnosis

Information from posts from 85 individuals with bipolar disorder who reported following a ketogenic diet was analysed further to seek information that would confirm their self-reported bipolar disorder diagnosis. We considered that bipolar disorder was ‘very likely’ when a diagnosis by an attending physician/psychiatrist was mentioned and/or references were made to taking medications that are the standard treatments for bipolar disorder. We considered that bipolar disorder was ‘likely’ when the individual reported having bipolar disorder and mentioned symptoms typical of bipolar disorder.

### Plausibility of achieving ketosis

We considered that ketosis was ‘very likely’ to have been achieved when one of the following was reported: weight loss; adherence to the diet for >1 week; note of persistence with diet through the adaption period; specific mention of adherence to a high fat, low carbohydrate diet. We considered that ketosis was ‘likely’ when the individual reported adherence to the diet.

### Data analysis

Frequencies of response classifications by dietary intervention together with 95% confidence intervals were calculated based on the agreed classifications of the two independent reviewers. Differences in response categories between those noting use of ketogenic diet versus those using other diets (omega-3 enriched or vegetarian) were calculated and χ² tests used to identify statistically significant differences.^15^ Since there were three *a priori* hypotheses being tested a *P*-value threshold of 0.017 (0.05/3 with Bonferroni correction) was applied.

As there may be a reporting bias, with those who did not find positive results being less likely to post about this, we compared reports with those associated with other dietary interventions that have been proposed to have mood stabilising effects and which are reported in bipolar blogs.

The primary research hypotheses that were defined *a priori* were:

- that the frequency of reports of mood stabilisation would be greater in those reporting following a ketogenic diet;
- that the frequency of reports of increased energy would be greater in those reporting following a ketogenic diet;
- that the frequency of reports of reduced anxiety would be greater in those reporting following a ketogenic diet.

All other research hypotheses were secondary and were developed *post hoc* after viewing the data and were regarded as exploratory and interpreted in this context.

#### Ethical approval

The ethical review procedures of the Usher Institute of Population Health Sciences and Informatics, University of Edinburgh were followed. A self-audit checklist for Level 1 Ethical Review was submitted and accepted. We considered that data on open public internet forums represented consent to access these data. As we did not know the identity of the individuals who posted, were not proposing to contact any individuals and we would be presenting data only in a broad summary format with no identifying details we considered there to be no issues of data confidentiality. In addition, we attempted to follow the principles contained in a recent document^16^ and hence have not included specific details of the online forums studied nor given quotations from the forums to avoid the risk of identification through online search engines.

## Results

Our primary hypothesis was that there would be an increased level of reporting of remission/significant mood stabilisation/some mood stabilisation among those adhering to a ketogenic diet compared with the two ‘control’ dietary regimens. We identified 165 posts relating to a ketogenic diet, 73 related to omega-3 supplementation and 21 related to a vegetarian diet. As there were only 21 posts related to a vegetarian diet we combined the responses to either vegetarian or omega-3 supplemented diets in the results below. Despite online speculation and discourse on possible benefit, there is to our knowledge, no credible evidence of an association between a vegetarian diet and bipolar disorder whereas there exists a body of published research on omega-3 supplementation that supports a possible association so the data is also provided separately by type of diet.

### Study 1: analysis of a large bipolar disorder online forum

#### Ketogenic diet

Table 1 presents details of reported improvement in mood stabilisation by category of improvement.

**Table 1:** Bipolar disorder online forum (study 1): *n* (%) of posts for dietary interventions categorised by reported level of change in mood stabilisation

	*n* (%, 95% CI)	
	Ketogenic diet (151 posts)	Omega-3 supplementation or vegetarian diet (40 posts)
Remission of symptoms	18 (11.9, 7.2–18.2)	1 (2.5, 0.1–13.2)
Significant mood stabilisation	69 (45.7, 37.6–54.0)	3 (7.5, 1.6–20.4)
Some mood stabilisation	40 (26.5, 19.6–34.3)	15 (37.5, 22.7–54.2)
No effect	16 (10.6, 6.2–16.6)	17 (42.5, 27.0–59.1)
Mood destabilisation	8 (5.3, 2.3–10.2)	4 (10.0, 2.8–23.7)
Significant mood destabilisation	0 (−)	0 (−)
Hospital admission/care-seeking	0 (−)	0 (−)

#### Ketogenic diet compared with other diets (vegetarian and omega-3 supplemented diets)

As there may be a reporting bias with those who did not find positive results being less likely to post about this, we compared reports with those associated with other dietary interventions that have been proposed to have mood stabilising effects and that are reported in blogs about bipolar disorder.

There were a higher proportion of reports of ‘any improvement in mood stabilisation’ with a ketogenic diet compared with the other two diets: 127 (84.1%, 95% CI 77.3–89.5) *v.* 19 (47.5%, 95% CI 31.5–63.9; χ² = 23.40, *P* < 0.0001). There were also a higher proportion of reports of ‘remission or significant improvement in mood stabilisation’ with a ketogenic diet compared with the other two diets: 87 (57.6%, 95% CI 49.3–65.6) *v.* 4 (10.0%, 95% CI 2.8–23.7; χ² = 28.57, *P* < 0.0001). There was no difference in the reporting of deterioration in mood stabilisation: 8 (5.3%, 95% CI 2.3–10.2) *v.* 4 (10.0%, 95% CI 2.8–23.7; χ² = 1.18, *P* = 0.3).

There were a substantially higher proportion of reports of ‘symptom remission’ with a ketogenic diet compared with the other two diets: 18 (11.9%, 95% CI 7.2–18.2) *v.* 1 (2.5%, 95% CI 1.0–13.2; χ² = 3.1, *P* = 0.08) but this did not reach statistical significance, likely due to the relatively small number of reports.

When looking in detail at the posts on a ketogenic diet it is clear that some experienced a definite effect within 3–7 days of starting the diet. There are reports of some mood destabilisation in a few (5.3%, 95% CI 2.3–10.2) individuals but this is not significantly different to the level reported with the other two diets (10.0%, 95% CI 2.8–23.7).

### Study 2: analysis of other online forums

#### Ketogenic diet compared with other diets (vegetarian and omega-3 supplemented diets)

There were also a higher proportion of reports of ‘remission or significant improvement in mood stabilisation’ with a ketogenic diet compared with the other two diets: 6 (42.9%, 95% CI 17.7–71.1) *v.* 8 (14.8%, 95% CI 6.6–27.1; χ² = 5.3, *P* = 0.021). There was no difference in the reporting of deterioration in mood stabilisation (see Table 2). The only two reports of ‘symptom remission’ were with a ketogenic diet compared with 0/54 with the other two diets.

**Table 2:** Other online forum (study 2): total number of posts for dietary interventions categorised by level of change in mood stabilisation

	*n* (%, 95% CI)	
	Ketogenic diet (14 posts)	Omega-3 supplementation or vegetarian diet (54 posts)
Remission of symptoms	2 (14.3, 1.8–42.8)	0 (−)
Significant mood stabilisation	4 (28.6, 8.4–58.1)	8 (14.8, 6.6–27.1)
Some mood stabilisation	8 (57.1, 28.9–82.3)	16 (29.6, 18–43.6)
No effect	0 (−)	15 (27.8, 16.5–41.6)
Mood destabilisation	0 (−)	3 (5.6, 1.2–15.4)
Significant mood destabilisation	0 (−)	0 (−)
Hospital admission/care-seeking	0 (−)	0 (−)

### Combined data-set

See Table 3 for the combined data-set (data for each diet group separately can be found in Table 4). We found that across the two studies 20 of 165 (12.1%, 95% CI 7.6–18.1) following a ketogenic diet compared with 1 of 94 (1.1%, 95% CI 0.0–5.8; χ² = 9.69, *P* = 0.002) reported ‘remission of symptoms’. Thus, a significantly higher proportion of those on a ketogenic diet reported extended periods of remission of symptoms. More than 95% of these reports of ‘remission of symptoms’ came from the ketogenic diet group. Some of these posts included those that reported details of very significant improvements, which had a significant impact on the quality of life of those reporting them.

**Table 3:** Combined analysis of all data: total number of posts for dietary interventions categorised by level of change in mood stabilisation

	*n* (%, 95% CI)	
	Ketogenic diet (165 posts)	Omega-3 supplementation or vegetarian diet (94 posts)
Remission of symptoms	20 (12.1, 7.6–18.1)	1 (1.1, 0.0–5.8)
Significant mood stabilisation	73 (44.2, 36.5–52.2)	13 (13.8, 7.6–22.5)
Some mood stabilisation	48 (29.1, 22.3–36.7)	38 (40.4, 30.4–51.0)
No effect	16 (9.7, 5.6–15.3)	34 (36.2, 26.5–46.7)
Mood destabilisation	8 (4.8, 2.1–9.3)	8 (8.5, 3.7–16.1)
Significant mood destabilisation	0 (−)	2 (2.1, 3.0–7.5)
Hospital admission/care-seeking	0 (−)	0 (−)

**Table 4:** Data from study 1 forum + other forums: total number of posts for dietary interventions categorised by level of effect in mood stabilisation

	*n* (%)		
	Ketogenic diet (165 posts)	Vegetarian diet (21 posts)	Omega-3 supplementation (73 posts)
Remission of symptoms	20 (12.1)	1 (4.8)	1 (1.4)
Significant mood stabilisation	73 (44.2)	5 (23.8)	10 (13.7)
Some mood stabilisation	48 (29.1)	2 (9.5)	28 (38.4)
No effect	16 (9.7)	7 (33.3)	27 (37)
Mood destabilisation	8 (4.8)	2 (9.5)	7 (9.6)
Significant mood destabilisation	0 (−)	0 (−)	0 (−)
Hospital admission/care-seeking	0 (−)	0 (−)	0 (−)

There were a higher proportion of reports of substantial improvement in mood stabilisation (i.e. reports of significant mood stabilisation or symptom remission) with a ketogenic diet compared to other diets: 93/165 (56.4%, 95% CI 48.4–64.1) *v.* 14/94 (14.9%, 95% CI 8.4–23.7). Thus the odds ratio for substantial improvement in mood stabilisation with ketogenic diet was 7.4 (95% CI 3.8–14.1 *P* < 0.0001). There were a higher proportion of reports of ‘any improvement in mood stabilisation’ with a ketogenic diet compared with the other two diets: 141 (85.5%, 95% CI 79.1–90.5) *v.* 52 (55.3%, 95% CI 44.7–65.6; χ² = 28.67, *P* < 0.0001). There were also a higher proportion of reports of ‘remission or significant improvement in mood stabilisation’ with a ketogenic diet compared with the other two diets: 93 (56.4%, 95% CI 48.4–64.1) *v.* 14 (14.9%, 95% CI 8.4–23.7; χ² = 42.37, *P* < 0.0001). There was no difference in the reporting of deterioration in mood stabilisation.

There were a higher proportion of reports of ‘any improvement in mood stabilisation’ and of ‘remission or significant improvement in mood stabilisation’ with a ketogenic diet compared with the omega-3 supplemented diet and with the vegetarian diet (see Table 3).

There were reports of mood destabilisation associated with a ketogenic diet in 8 of 165 (4.8%, 95% CI 2.1–9.3) of individuals. However, this was not statistically significantly different to the frequency of reports of destabilisation on other diets (8/94, 8.5%, 95% CI 3.7–16.1; χ² = 3.50, *P* = 0.06). Some of these reports noted that the destabilisation occurred after discontinuing medication after achieving positive results with a ketogenic diet.

### Plausibility of diagnosis

From the information given in the posts of 85 individuals we judged that 64 (75.3%, 95% CI 64.7–84.0) were very likely (*n* = 49) or likely (*n* = 15) to have a diagnosis of bipolar disorder. Of 24 individuals who reported a subtype of bipolar disorder, 16 (66.7%, 95% CI 44.7–84.4) mentioned type II and 8 (33.3%, 95% CI 15.6–55.3) reporting having type I.

### Plausibility of achieving ketosis

From the reports of experience with a ketogenic diet we judged that of 85 individuals 79 (92.9%, 95% CI 85.3–97.4) were very likely (*n* = 52) or likely (*n* = 27) to have achieved a state of ketosis. Nineteen (22.4%, 95% CI 14.0–32.7) individuals noted that there was an adaptation period before positive effects of the diet were found. The most common problems adhering to the diet were difficulties in initially adapting to the diet in nine (10.6%, 95% CI 5.0–19.2) and a return of bipolar disorder symptoms when carbohydrates were reintroduced into the diet in six (7.1%, 95% CI 2.6–14.7).

### Reported range and strength of effects of ketogenic diet

The following positive effects associated with adoption of a ketogenic diet were reported in 85 individuals: improved mood stability in 55 (65%); fewer episodes of depression in 35 (41.2%, 95% CI 30.6–52.4); improved clarity of thought and speech in 24 (28.2%, 95% CI 19.0–39.0); increased energy in 22 (25.9%, 95% CI 17.0–36.5); reduced anxiety/panic attacks in 17 (20.0%, 95% CI 12.1–30.1); fewer episodes of mania in 11 (12.9%, 95% CI 6.6–22.0); improved sleep in 7 (8.2%, 95% CI 3.4–16.2); improved control of actions in 7 (8.2%, 95% CI 3.4–16.2); improved memory in 2 (2.4%, 95% CI 0.3–8.2). When comparing those whose achievement of ketosis was rated ‘very likely’ versus ‘likely’ or ‘unclear’ then there was a tendency for improved energy (17/52, 32.7%, 95% CI 20.3–47.1 *v.* 4/32, 12.5%, 95% CI 3.5–29.0; χ² = 4.26, *P* = 0.039) and improved mood stabilisation (38/52, 73.1%, 95% CI 59.0–84.4 *v.* 17/32, 53.1%, 95% CI 34.7–70.9); χ² = 3.46, *P* = 0.063).

Twenty-two (25.9%, 95% CI 17.0–36.5) also mentioned weight loss that was generally reported as a positive feature and there was a report of improvement in acne. In 33 reports in which further information about effect on mood was given 26 (78.8%, 95% CI 61.1–91.0) described this as mood stabilisation and 7 (21.2%, 95% CI 9.0–38.9) as mood elevation.

Forty-one individuals reported information from which it was possible to understand the duration of reported benefit. In 16 (39.0%, 95% CI 24.2–55.5) of these reports this was greater than 6 months and in 10 (24.4%, 95% CI 12.4–40.3%) it was greater than 12 months. Other reports of benefit were for periods of up to 1 month (5 posts), from 1 up to 3 months (14 posts) or from 3 to 5 months (6 posts).

Many of the forum posts reported clear histories of bipolar disorder and receiving medical treatment, including periods of admission to hospital. Reports included those of dramatic reductions of symptoms to an ‘asymptomatic’ condition, or ‘cure,’ or ‘complete reduction of all symptoms,’ or ‘life-changing’ levels of relief. Reports also detailed the ability to perform important life tasks that were previously not possible. Some reported mood stability exceeding that previously achieved on medication or leading to reductions in or cessation of previous medication. Length of improved mood stabilisation was often reported to be for months to years (with 8 years being the longest period reported).

## Discussion

### Main findings

This observational analytic study of bipolar disorder online forums provides some preliminary support for the hypothesis that a ketogenic diet can have beneficial effects on mood stabilisation that can be long lasting. The study is based on self-reports and individual experience and so bipolar disorder diagnosis and mood response cannot be confirmed by expert review and also online reports may be subject to considerable reporting bias. Nevertheless, reports of patient experience are an important health outcome and are valid in themselves. Furthermore, this controlled study sought to minimise biases by adopting a control group, having independent review of text comments, adopting partial masking of assessment and replicating findings in a separate study.

In assessing forum reports we only included those reports based on direct personal experience and excluded second-hand reports of experience of other people and reports in which people stated what they had read about or believed would happen. There was a higher level of improvement in mood stabilisation with a ketogenic diet compared with the omega-3 diet and vegetarian diets (and compared with a group of those on either of these ‘control diets’). The difference was particularly marked among those reporting ‘remission or significant improvement in mood stabilisation’ (56.4% *v.* 14.9%) and with remission of symptoms (12.1% *v.* 1.1%). The difference in the quality and detail in the reporting of improvements was striking with more detailed reports of benefits in the ketogenic diet group. There was a tendency for the improvement in mood stabilisation to be greater among those whose dietary report included details that suggested that they had achieved a ketotic state. These findings were not confined to one online forum community as data from the independent data mining of other online forums (study 2) yielded similar levels of reporting of mood stabilisation and replicated the study 1 forum reports of improved mood stabilisation associated with a ketogenic diet.

### Study limitations

There are a number of limitations in this study. Forum posts are subjective reports that may be influenced by media reports and peer factors. For this reason, we adopted a control group consisting of other proposed dietary interventions for bipolar disorder (omega-3 fatty acid supplementation and a vegetarian diet) but this may have not controlled adequately for these biases. Forum posts may be influenced strongly by previous (favourable or adverse) posts on a specific forum giving a ‘culture’ on that forum that favours certain types of response. We attempted to mitigate this possible effect by looking at posts across more than one forum. Our findings suggest that these reports of mood stabilisation with a ketogenic diet were not confined only to selected online forums. The bipolar disorder diagnosis was self-reported and may have been inaccurate in some cases. We made an assessment of our confidence in the diagnosis (with defined criteria) and found that the bipolar disorder diagnosis was ‘likely’ or ‘very likely’ in 75% of individuals who reported effects of ketogenic diet. This is a subjective assessment but based on explicit criteria (defined *a priori*) given in the paper. Adherence to the three diets under study was variable and challenging to assess from the reports. Nevertheless, we judged that the great majority (93%) of forum posts from those reporting a ketogenic diet were from individuals who had successfully achieved a state of ketosis. It is possible that some of the benefits reported were short term and, in a condition characterised by remissions and relapses, this might not give reliable information about true benefits. We assessed this by looking at reported duration of benefit and found this to be >6 months in about 40% of reports where information on duration was given. In addition, the magnitude and prolonged duration of the benefit noted by some participants suggests that this is not a minor transient benefit, at least for a significant proportion of individuals. However, these interpretations of the data may be incorrect in some cases. We present these findings as a hypothesis-generating study that clearly needs to be investigated further, ideally by means of an experimental study such as a clinical trial, as detailed above.

### Putative mechanisms

The suggested mechanisms of action of a ketogenic diet broadly fall under two broad categories: mechanisms of mitochondrial dysfunction and alterations of neuronal intracellular sodium and calcium.

#### Mitochondrial dysfunction

**Fig. 1:**
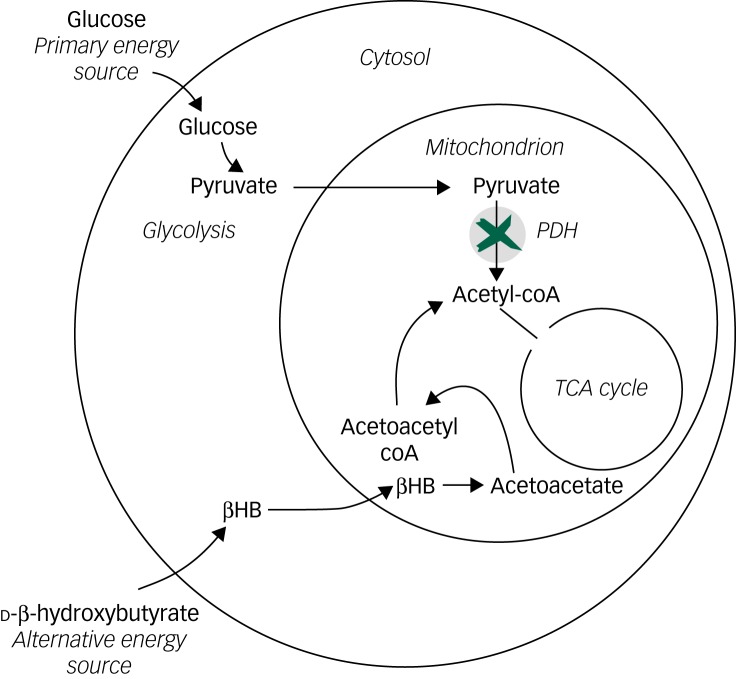
Simplified diagram of how ketone bodies such as d-beta-hydroxybutyrate may bypass a block in the link between glycolysis (pyruvate to acetyl coenzyme A (acetyl-coA)) in the cytoplasm and the tricarboxylic acid (TCA) cycle in mitochondrion.

PDH, pyruvate dehydrogenase; βHB, beta-hydroxybutyrate.

The evidence for mitochondrial dysfunction in bipolar disorder has been summarised by Kato^17^ and Kim *et al*.^10^ Impairment of oxidative phosphorylation and the tricarboxylic acid (TCA) cycle is a key component with increased levels of pyruvate from glycolysis suggesting an impaired ability to utilise pyruvate within the TCA cycle.^18^ In a state of ketosis, high plasma levels of ketones such as beta-hydroxybutyrate act as an alternative energy source and can supply acetyl coenzyme A (acetyl-coA) directly to the TCA cycle providing an alternative pathway to that from pyruvate (see Fig. 1). The reports of the positive effects of a ketogenic diet on mood stabilisation in bipolar disorder are consistent with a reduced formation of acetyl-coA from pyruvate that is bypassed by this alternative source of acetyl-CoA from ketones. This, in turn, implies reduced activity of mitochondrial pyruvate dehydrogenase (PDH) or reduced carriage of intracellular pyruvate transport across the mitochondrial membrane by mitochondrial carrier proteins (MPC) (see Fig. 1). Metabolomic studies have demonstrated disruption of the TCA cycle in bipolar disorder and have reported increased levels of pyruvate^18^ but are not clear about the biochemical antecedents of these findings. Our study findings support the hypothesis that improved mood stabilisation is associated with a ketogenic diet. If true, this provides new information that may help understand the nature of the underlying biochemical mechanism and is consistent with reduced activity of PDH and/or MPC being important in some patients with bipolar disorder.^19^ To investigate this hypothesis further would require further detailed metabolomics studies of plasma and cerebrospinal fluid of patients with bipolar disorder, including those who are adhering to a ketogenic diet and have high levels of plasma beta ketones.

#### Reduction of intracellular sodium and calcium

Bostock *et al* point to reduction of intracellular sodium and calcium as an important mechanism of action for mood stabilisation on a ketogenic diet.^20^ Phelps *et al*^11^ notes that successful mood stabilising medications reduce intracellular sodium in an activity-dependent manner and that this can also be achieved through acidification of the blood on a ketogenic diet. Phelps and colleagues hypothesise that in the ketotic state extracellular protons in acidified blood are exchanged with intracellular sodium thereby preventing excessive intracellular sodium accumulation. It is also possible that reduced intracellular sodium occurs as a downstream effect of restored mitochondrial function. El-Mallakh notes that conditions of reduced mitochondrial ATP production may lead directly to altered sodium and calcium dynamics in neurons mediated by reduced Na,K–ATPase activity.^21^ This hypothesis may provide insight into a mechanism underlying both the mounting evidence for mitochondrial dysfunction and the altered sodium/calcium levels typically observed.

### Implications

Despite inherent limitations of observational data based on self-reports posted online this controlled observational analytic study of bipolar disorder online forums gives support to the hypothesis that a ketogenic diet can have beneficial effects on self-reported mood stabilisation.^22^ These findings are consistent with a mitochondrial dysfunction component to bipolar disease aetiology. The effect could be mediated by ketones bypassing a block in the linkage between glycolysis and the TCA cycle because of reduced activity of pyruvate dehydrogenase or mitochondrial pyruvate carriage. Ketone ester compounds are now available that replicate the high ketonaemia found in those following a ketogenic diet.^23^ Adoption of a ketogenic diet or use of ketone esters (if these are shown to have an adequate safety profile for such use) could be investigated further for their potential use in clinical trials to investigate this hypothesis further.

## Supplementary material

For supplementary material accompanying this paper visit http://dx.doi.org/10.1192/bjo.2019.49. click here to view supplementary material
