# Supplementary material for: Ketosis and bipolar disorder: controlled analytic study of online reports
Source: BJPsych Open. 2019 Jul 4;5(4):e58. doi: 10.1192/bjo.2019.49 (PMC6620566; doi:10.1192/bjo.2019.49)
Supplement: Supplementary file 1 [file S2056472419000498sup001.docx]

**Supplementary File 1: *A priori* criteria/definitions adopted for classifying online entries:**

**Diagnosis: (1) Very likely (2) Likely (3) Unclear (4) No Diagnosis**

1. Very Likely - user references the diagnosis of an attending physician/psychiatrist and/or references taking medications which are the standard treatments for BD.
2. Likely - user identifies as having BD and mentions common symptoms.
3. Unclear - user mentions mental health issues with no specific mention of BD or its symptoms.
4. No Diagnosis - user mentions that they have not been officially diagnosed.

**Type of Bipolar Disorder Type (1) BD Type 1 (2) BD Type 2 (3) Cyclothymia**

- Criteria: specific mention in text of Bipolar Type 1, Bipolar Type 2 or Cyclothymia.

**Energy Improvement: (1) Yes (2) No**

- Report of increase on increase in energy/wakefulness as a direct result of adoption of the diet.

**Depression Improvement: (1) Yes (2) No**

- Report of improvement in depression or happier mood as a direct result of adoption of the diet.

**Mania Improvement: (1) Yes (2) No**

- Report of reduced manic episodes as a direct result of adoption of the diet.

**Mood Stability Improvement: (1) Yes (2) No**

- Report of improved mood stability or less variation of mood as a direct result of adoption of the diet.

**Anxiety and Panic Attack: Improvement: (1) Yes (2) No**

- Report of reduction of anxiety and/or panic attacks as a direct result of adoption of the diet. Or increased calmness.

**Memory Improvement: (1) Yes (2) No**

- Report of improved memory function as a direct result of adoption of the diet.

**Thought/Speech Clarity Improvement: (1) Yes (2) No**

- Report of clearer thinking or speech as a direct result of adoption of the diet.

**Sleep Improvement: (1) Yes (2) No**

- Report of improved quality/length of sleep a direct result of adoption of the diet.

**Control of Actions: Improvement: (1) Yes (2) No**

- Report of positive behaviour change as a direct result of adoption of the diet.

**Weight Loss: (1) Yes (2)**

- Report of weight loss as a direct result of adoption of the diet.

**Skin Improvement: (1) Yes (2) No**

- Report of improved skin condition as a direct result of adoption of the diet.

**Duration of Mood/Energy Benefit:**

- (0) 1 - 4 weeks (1) 1 Month- 3 Months (2) 3 - 6 Months (3) 6 - 9 Months (4) 9 - 12 Months (5) Over a year

**Mood Elevation or Stabilization (1) Elevation (2) Stabilization (3) Depression**

1. Elevation - Report of increased happiness only.
2. Stabilization - Report of reduced depression and mania.
3. Depression - More depression.

**Ketosis Achieved: (1) Very likely (2) Likely (3) Unclear (4) Unlikely (5) Very Unlikely**

1. Very likely - Report of one of the following:
   1. Weight loss. Adherence over 1 week.
   2. Persistence through adaption period.
   3. Specific mention of adherence to high fat, low carbohydrate diet.
2. Likely - Report of adherence to diet.
3. No specific information about diet.
4. Adherence to diet less than 3 days.
5. Adherence to diet less than 3 days or misunderstanding of a correctly formulated Ketogenic diet.

**Adaption Period (1) Yes (2) No**

- Symptoms of adaptation to the diet note (initial low energy, mood and/or hunger which then improves.)

**Medication:**

- Valproic Acid (2) Carbamezapine (3) Lithium (4) Lamictal (5) Latuda (6) Cipralex (7) Escitalopram (8) Clonazepam (9) Klonopin (10) Xanax (11) Latuda (12) Olanzapine

**Problems Reported (1) Difficult Adaption (2) Hunger (3) Diet Adherence (4) Return of Symptoms with Re-introduction of Carbohydrates**

1. Initial low energy and/or mood which then improves.
2. Difficulty with hunger
3. Difficulty in maintaining the diet.
4. Return of BD symptoms when carbohydrates are reintroduced to diet.
